# Tenogenically Induced Allogeneic Mesenchymal Stem Cells for the Treatment of Proximal Suspensory Ligament Desmitis in a Horse

**DOI:** 10.3389/fvets.2015.00049

**Published:** 2015-10-22

**Authors:** Aurélie Vandenberghe, Sarah Y. Broeckx, Charlotte Beerts, Bert Seys, Marieke Zimmerman, Ineke Verweire, Marc Suls, Jan H. Spaas

**Affiliations:** ^1^Global Stem Cell Technology, ANACURA Group, Evergem, Belgium; ^2^Equine Veterinary Pratice Dr. Suls, Nederweert, Netherlands; ^3^Equine Diagnostic Center, Meldert-Lummen, Belgium

**Keywords:** allogenic, stem cells, tendon, suspensory, horse

## Abstract

Suspensory ligament injuries are a common injury in sport horses, especially in competing dressage horses. Because of the poor healing of chronic recalcitrant tendon injuries, this represents a major problem in the rehabilitation of sport horses and often compromises the return to the initial performance level. Stem cells are considered as a novel treatment for different pathologies in horses and humans. Autologous mesenchymal stem cells (MSCs) are well known for their use in the treatment of tendinopathies; however, recent studies report a safe use of allogeneic MSCs for different orthopedic applications in horses. Moreover, it has been reported that pre-differentiation of MSCs prior to injection might result in improved clinical outcomes. For all these reasons, the present case report describes the use of allogeneic tenogenically induced peripheral blood-derived MSCs for the treatment of a proximal suspensory ligament injury. During conservative management for 4 months, the horse demonstrated no improvement of a right front lameness with a Grade 2/5 on the American Association of Equine Practitioners (AAEP) scale and a clear hypo-echoic area detectable in 30% of the cross sectional area. From 4 weeks after treatment, the lameness reduced to an AAEP Grade 1/5 and a clear filling of the lesion could be noticed on ultrasound. At 12 weeks (*T*_4_) after the first injection, a second intralesional injection with allogeneic tenogenically induced MSCs and platelet-rich plasma was given and at 4 weeks after the second injection (*T*_5_), the horse trotted sound under all circumstances with a close to total fiber alignment. The horse went back to previous performance level at 32 weeks after the first regenerative therapy and is currently still doing so (i.e., 20 weeks later or 1 year after the first stem cell treatment). In conclusion, the present case report demonstrated a positive evolution of proximal suspensory ligament desmitis after treatment with allogeneic tenogenically induced MSCs.

## Introduction

Suspensory ligament injuries are a major issue in equine orthopedics. It is indicated in both elite and non-elite dressage horses to have a higher risk compared to horses undertaking general-purpose exercise or doing other disciplines (1). Commonly, it is the proximal aspect of the suspensory ligament that is affected in these sport horses (2).

The limited healing of tendon tissue, with the formation of scar tissue, has been attributed to the low number of residing cells in relation to the extensive volume of the extracellular matrix. The reduced healing capacity of tendons and ligaments is a major problem in the rehabilitation of sport horses and leads to chronic injuries with a poor prognosis (3, 4). Indeed, these tendinous lesions often compromise the return of the horse to the initial performance level.

Therapies for suspensory ligament pathologies consist of conservative management, regenerative therapies, and surgical therapies. Conservative management aims at promoting the optimal healing of an acutely damaged tendon by medical treatments, rest, cold applications, corrective shoeing, and controlled exercises (5–8). For chronic tendinopathies, eccentric tendon training, extracorporeal shockwave therapy, and surgical therapy are indicated (8). Surgical therapy consists of a fasciotomy, with neurectomy of the deep branch of the palmar or the plantar nerve, or a suspensory desmoplasty (surgical fasciotomy and splitting) (9–11).

Recent regenerative therapies, containing growth factor-based therapies and cell therapies, report an improvement of tendon healing and reduction of recidives (2, 8, 12). For safety (no calcifications) and efficacy (functional recovery) reasons, it has been reported that inducing stem cells toward a tenogenic phenotype is a valuable alternative for conservative treatments (12) and adding platelet-rich plasma (PRP) generates superior effects in comparison to both treatments separately for the treatment of degenerative joint disease (13). Moreover, allogeneic PRP and cell-based therapies represent a valuable alternative for autologous treatments because of the immediate availability and ease to use in field circumstances. Indeed, different groups report a safe intradermal, intra-articular, and intravenous application of allogeneic mesenchymal stem cells (MSCs) in a total of more than 500 horses in all studies together (13–17). Although several studies report the safe and effective use of allogeneic MSCs in equine tendons (12, 18–20), only limited information on tenogenically induced allogeneic MSCs and allogeneic PRP for the treatment of chronic tendon lesions is currently available (21).

For all the aforementioned reasons, this case report describes the treatment of a chronic recalcitrant proximal suspensory ligament desmitis with allogeneic tenogenically induced peripheral blood-derived MSCs in combination with allogeneic PRP.

## Case Details

### History

A 9-year-old, KWPN stallion, intended for dressage work at higher level (sub Grand Prix), was diagnosed with a lesion at the lateral border of the proximal aspect of the suspensory ligament. The horse was treated 4 months ago with non-steroidal anti-inflammatory drugs (NSAIDs) and a single intralesional injection of PRP combined with a controlled rehabilitation program. This consisted of 2 weeks of hand walking and daily cooling of the leg with a soft protecting resting bandage during the night, followed with 1 week of walk and trot on a straight line. Afterwards, gradual walking and trotting exercises were performed for 3 weeks starting with 2 min trot a day with an increase of 2 min every 2 days up to 20 min trotting per day. Treatments remained clinically and ultrasonographically unresponsive.

### Clinical Examination and Diagnostic Analgesia

Four months after the initial diagnosis and first treatment program, the horse was presented for an orthopedic examination. At the initial clinical examination, as well as 4 months later (i.e., before the first stem cell injection), the horse showed a right front lameness of 2/5 on the American Association of Equine Practitioners (AAEP) scale (Table 1). The lameness was consistent in degree in straight lines. When trotting on the circle to the left on a soft surface, the right front lameness was more pronounced with a marked reduced cranial phase of the stride. Passive flexion of the lower part of the right front limb exacerbated the lameness.

**Table 1:** **AAEP lameness scale**.

Grade degree of lameness (on 5)	
0	Lameness not perceptible under any circumstances
1	Lameness is difficult to observe and is not consistently apparent, regardless of circumstances
2	Lameness is difficult to observe at a walk, or when trotting in a straight line, but consistently apparent under certain circumstances
3	Lameness is consistently observable at a trot under all circumstances
4	Lameness is obvious at a walk
5	Lameness produces minimal weight bearing in motion and/or at rest or a complete inability to move

Several diagnostic anesthesia’s of the right front limb, each with 2% Mepivacaine hydrochloride solution, were performed. A distal digital nerve block, an abaxial nerve block and a low four point nerve block were negative. A nerve block of the deep branch of the lateral palmar nerve almost completely abolished the right front lameness to a score of 0.5/5 on the AAEP scale.

### Diagnostic Imaging

#### Radiographic Examination

Dorsopalmar, lateromedial, and oblique radiographic projections of the right proximal metacarpal region were obtained and compared with the contralateral limb. No abnormal findings were noticed.

#### Computed Tomography

**Figure 1:**
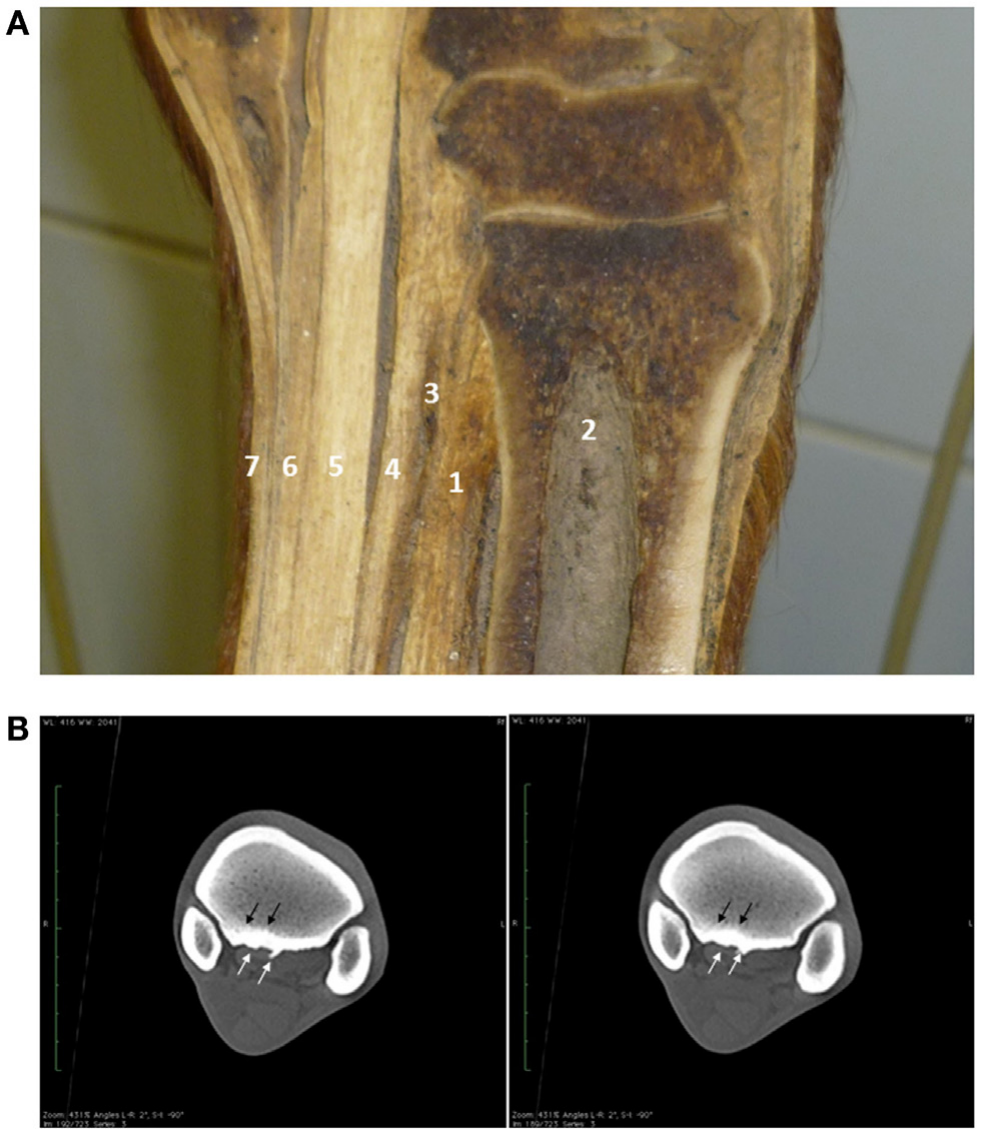
**Longitudinal anatomical section of the proximal metacarpus showing the origin of the proximal third interosseus muscle (suspensory ligament) over the third metacarpal bone and distal carpus**. 1 – suspensory ligament; 2 – third metacarpal bone; 3 – deep palmar metacarpal vascular anastomosis; 4 – accessory ligament of the deep digital flexor tendon; 5 – deep digital flexor tendon; 6 – superficial digital flexor tendon; 7 – skin **(A)**. Computer tomography demonstrated a mild to moderate bone remodeling (white arrows) and sclerosis (black arrows) at the proximal aspect of the third metacarpal bone at the insertion of the suspensory ligament **(B)**.

In mutual agreement with the owner, computed tomography was performed in order to further evaluate the palmar aspect of the proximal third metacarpal bone (Figure 1A). Contrast angiography was not performed in this horse.

Mild to moderate bone remodeling of the palmar third metacarpal bone was observed. This was more pronounced medially, with the presence of a large osteophyte axially (Figure 1B). Moreover, a moderate sclerosis at the medial aspect of the proximopalmar third metacarpal bone was present (Figure 1B).

#### Ultrasound Examination

A 7.5-MHz linear ultrasound probe was used to evaluate the palmar aspect of the right metacarpal region. A complete examination of the suspensory ligament was performed during each ultrasound examination with both transverse and longitudinal scans. The scoring system was adapted from a previous study by Beerts et al. (21) and can be found in Table 2.

**Table 2:** **The scoring system used to evaluate and compare the ultrasound images in the present report**.

Score	Echogenicity	Fiber pattern/alignment	Size of ligament
0	Anechoic area (central core lesion)	Lacking of parallel pattern acute injury (hemorrhage), 0–25% FA	Enlarged width and thickness
1	Lesion site starting to fill with presence of hypo-echoic areas, and moderate diffuse decrease in echogenicity	Lacking of nice parallel pattern, 0–25% FA	Enlarged width and thickness
2	Lesion site gradually filling with presence of multiple areas with decreased echogenicity	Increased parallel pattern, 25–50% FA	Enlarged width and thickness
3	Demarcation between injured and uninjured tendon less distinct, hypo-echoic areas are remaining	Increased parallel pattern, 25–50% FA	Mild enlarged width and thickness
4	Hardly any demarcation between injured and healthy tissue, faint signs of hypo-echoic areas	Close to total fiber alignment, 75–100% FA	Almost no enlarged width and thickness
5	Echogenicity (almost) identical to contralateral ligament	Close to total fiber alignment, 75–100% FA	No enlarged width and thickness

*Different parameters, such as echogenicity, fiber pattern/alignment (FA) grading, and size (width and thickness) of the ligament, were considered and compared to the contralateral limb*.

**Figure 2:**
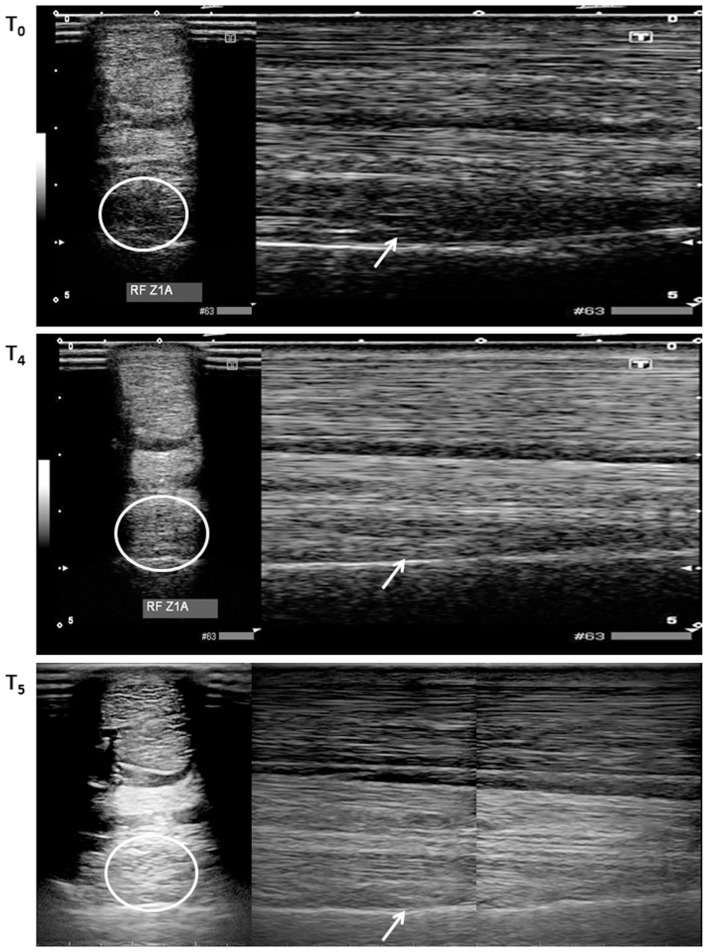
**Transverse (encircled lesion area) and longitudinal (arrow in lesion area) ultrasound images of the proximal aspect of the lateral lobe of the suspensory ligament performed at *T*_0_, *T*_4_ (12 weeks), and *T*_5_ (16 weeks)**. At *T*_0_, a clear hypo-echoic area could be noticed on both ultrasounds. At *T*_4_, only limited remaining hypo-echoic areas were noticed, whereas this clearly reduced at *T*_5_.

At the proximal aspect of the lateral lobe of the suspensory ligament, a focal hypo-echogenic area with an abnormal fiber alignment was present and consisted of approximately 30% of the cross sectional area of the suspensory ligament. In comparison with the clinically sound contralateral limb, the proximal aspect of the suspensory ligament was enlarged with a poor demarcation of the dorsal margin (Figure 2).

### Treatment

Four weeks after the computed tomography examination, an intralesional injection with allogeneic tenogenically induced MSCs and PRP (from Global Stem Cell Technology) in ­combination with NSAIDs was performed by an equine orthopedic ­specialist. Both tenogenically induced MSCs and PRP were prepared from a 6-year-old German Warmblood horse and characterized as previously described (12, 22).

Briefly, 50 ml of peripheral blood was collected from the *vena jugularis* in ethylenediaminetetraacetic acid (EDTA) tubes for MSC isolation. Subsequently, the blood was centrifuged at 1000 × *g* for 20 min and the buffy coat was collected and diluted 1:2 with Hanks’ Balanced Salt Solution (HBSS, Life Technologies). This suspension was gently layered on an equal amount of Percoll (GE Healthcare). After different washing steps with HBSS, 20 million peripheral blood mononuclear cells (PBMCs) were seeded per *T*_75_ flask and cultured until 60% confluency was reached. Trypsinization was performed with 0.25% trypsin–EDTA until passage 6 (P6) was reached. Going to P7, 0.5 million MSCs were seeded in a T75 flask and tenogenically induced by supplementing tendogenic growth factors to the expansion medium until 80% confluency was reached, and tenogenic differentiation markers were upregulated (Gomiero et al., in review). A higher passage was used to allow upscaling in future cell manufacturing processes, which is considered to be possible thanks to the phenotypic stability of PB–MSCs from P5 up to P10 (23, Gomiero et al., in review). After trypsinization, all the cells were resuspended in 1 ml of DMEM low glucose (Life Technologies) with 10% of dimethyl sulfoxide (DMSO, Sigma). The samples were stored in −80°C until all quality controls were completed. The latter consisted of sterility testing and flow cytometry assessment for (stem) cell markers, as previously reported (22, 23).

For PRP preparation, 300 ml of peripheral blood was taken in a citrate phosphate dextrose adenine-1 (CPDA-1) single blood bag (Terumo^®^) from the same donor. Platelets were purified by means of subsequent centrifugation steps, as previously reported (24) until more than 80% platelets were obtained at a concentration of more than 100 × 10^6^ platelets in 1 ml. Cells and PRP were shipped on dry-ice for clinical application. After trypan blue staining, it became clear that more than 75% of the tenogenically induced MSCs were viable upon injection.

The horse was sedated with 0.04 mg/kg detomidine (Domosedan^®^) and 0.1 mg/ml butorphanol (Turbogesic^®^) intravenously, and the treatment site was aseptically prepared. A 7.5 MHz linear ultrasound probe was covered with a sterile glove to perform the ultrasound-guided intralesional injection. A sterile 21G needle was introduced into the lesion and connected to the syringe with the tenogenically induced MSCs (1 ml) and PRP (1 ml). After the treatment, a soft protective bandage was applied and left on for 1 day, followed by the exact same rehabilitation program as described above for the first treatment.

A second intralesional injection with allogeneic tenogenically induced MSCs and PRP was performed 12 weeks after the first injection in order to stimulate the healing after stagnation.

### Outcome

#### Clinical Assessment

Clinical evaluation was performed 4 weeks before injection (*T*_0_), and at 4-week intervals after the first injection (*T*_1_ = 0 weeks, *T*_2_ = 4 weeks, *T*_3_ = 8 weeks, *T*_4_ = 12 weeks, *T*_5_ = 16 weeks). A second injection was performed 12 weeks after the first injection at *T*_4_. Clinical evaluation was performed according to the AAEP scoring system by the same team of veterinarians.

At *T*_0_, the horse showed a right front lameness with a Grade 2/5 consistent in degree in straight lines and more pronounced when trotting on a circle to the left on a soft surface (Table 3). At *T*_1_, 4 weeks after the diagnosis, the horse was treated by a first intralesional injection with the combination of peripheral blood-derived tenogenically induced MSCs and PRP. At subsequent evaluation time point *T*_2_, the lameness was reduced to a right front lameness with a Grade 1/5 and an improvement in the cranial phase of the stride (Table 3). At *T*_3_ and *T*_4_, the lameness was graded 0–1/5, because it was inconsistently apparent and difficult to observe.

**Table 3:** **Evaluation of the proximal suspensory healing by clinical assessment and ultrasound examination at 4 weeks before the injection (*T*_0_), the day of injection (*T*_1_), and every 4 weeks up to week 16 (*T*_2–5_)**.

	*T*_0_	*T*_1_	*T*_2_	*T*_3_	*T*_4_	*T*_5_
Lameness score AAEP	2	2	1	0–1	0–1	0
Ultrasound score	1	1	2	3	3	4

A second intralesional injection with MSCs and PRP was performed at *T*_4_, 12 weeks after the first injection. At evaluation time point *T*_5_, 4 weeks after the second intralesional injection (i.e., 16 weeks after the first injection), the horse trotted completely sound under all circumstances tested (Table 3). Communication with the owner revealed that the horse started performing at previous level (sub Grand Prix) at 32 weeks after the first regenerative treatment and is currently still doing so (i.e., 20 weeks later or 1 year after the first stem cell treatment).

#### Ultrasound Examination

A score between 0 and 5 for the ultrasound images was given. At *T*_0_ and *T*_1_, the horse received a score 1, which corresponded with the initiation of lesion filling with the presence of hypo-echoic areas, a moderate diffuse decrease in echogenicity, lacking of a parallel fiber pattern and 0–25% fiber alignment (Figure 2). At *T*_2_, a score of 2 was given, which indicated a gradual filling of the lesion with the presence of multiple areas with decreased echogenicity, an increased parallel fiber pattern and 25–50% fiber alignment. At subsequent evaluation time points, *T*_3_ and *T*_4_, a score of 3 corresponded with a less distinct demarcation between injured and uninjured tendon with remaining hypo-echoic areas, an increased parallel fiber pattern, 25–50% fiber alignment and reduced width and thickness. At 4 weeks after the second injection (*T*_5_), a score of 4 was given, which indicated hardly any demarcation between injured and healthy tissue, faint hypo-echoic areas, an almost total fiber alignment and very mild increased thickness (Figure 2). An overview of the ultrasound scores can be found in Table 3.

## Discussion

Proximal suspensory ligament injuries are an important source of lameness in performance horses. Because of both soft tissue and bone components, the suspensory ligament has a complex anatomy, and therefore, diagnosis should be made by multiple modalities. A good diagnosis can be made by the combination of ultrasound examination and radiology, but often advanced imaging, such as nuclear scintigraphy, magnetic resonance imaging, and computed tomography, will be required to detect certain types of injury (25).

In this case report, diagnosis was made by ultrasound, radiology, and computed tomography. However, no contrast angiography was performed, which could have provided more information on lesion severity and perfusion. Indeed, contrast angiography would have allowed us to distinguish between the normal fat and muscle tissue at the origin of the suspensory ligament and a lesion of the ligament (26–28). This would have provided more detailed information on the extent of the soft tissue lesion and should be considered for diagnostic and treatment evaluation in future studies. Nevertheless, in the present report, computed tomography accurately identified new bone formation that was not fully revealed via radiology and ultrasound. In horses with a chronic or recurring lameness origination from the proximal aspect of the third metacarpal bone, new bone formation at the proximal region of insertion of the suspensory ligament, should be considered as a differential diagnosis (29).

Since it has been described that uninduced MSCs have the ability to form ectopic bone in calcified areas (30), the use of tenogenically induced MSCs represented a safe alternative in this case report. Nevertheless, larger studies comparing both treatment modalities will have to provide more insights in this matter. In addition, treatment after ultrasound-guided intralesional injection supports suspensory ligament healing and has a better prognosis, compared to conservative therapy, for returning to sport activity (2). In the present report, PRP and peripheral blood-derived tenogenically induced MSCs were injected together, as previously described by our group (12). Since the suspensory ligament injury was irresponsive to PRP treatment alone (cfr. Patient history) and no control group without PRP was used, no conclusions can be made concerning the necessity of PRP addition in order to obtain the desired clinical effect. However, for degenerative joint disease in horses, a substantial improvement of the clinical outcome could be demonstrated after PRP addition to allogeneic MSCs (13). This was probably attributed to the stem cell stimulating growth factors that are present in PRP. For this reason, the latter substance was added to the tenogenically induced MSCs. Further studies will provide more clarity on the effect of PRP on MSC-related therapies for tendon healing in horses.

It should be mentioned though that at 12 weeks after the first treatment, hypo-echoic areas with a discrete demarcation between injured and uninjured tendon were still present on the ultrasound images. This is in contrast with 11 out of 15 horses with suspensory ligament desmitis, which demonstrated only faint signs of hypo-echoic areas and hardly any demarcation between injured and healthy tissue at 6 weeks post injection (12). In this case study, the latter state was only reached at 4 weeks after the second injection (i.e., 16 weeks after the first injection). An explanation for the noticed discrepancy might be that the present case demonstrated new bone formation, which compromised the first treatment. Indeed, it has been previously reported that carpal osteophytes might result in tendon ruptures (31). Moreover, suspensory ligament desmitis has a poor prognosis in general (32, 33) and conservative management for 4 months did not result in any improvement in this case. Nevertheless, a second injection reactivated the healing and resulted in recovery. Since it has been demonstrated that autologous MSCs elicit a similar immune response than allogeneic MSCs in equine superficial digital flexor tendons, no immunogenic hypersensitivity was expected nor observed after repeated injections (18). In the present case with a chronic suspensory ligament injury, a second injection was warranted due to remaining hypo-echoic areas and abnormal fibern pattern. Although no reports are available describing the influence of repeated MSC injections on chronic tendon injuries, repeated MSC treatments of liver fibrosis (34) and cardiomyopathies (35, 36) resulted in significantly enhanced tissue repair and functionality in comparison to single dose treatments. In both organs, reduced fibrosis was at the basis of the noticed improvements and this was achieved by remodeling of the collagen network, which is a crucial element in chronic tendon injury as well (8).

In conclusion, a positive evolution of proximal suspensory ligament desmitis could be demonstrated after repeated injection with allogeneic tenogenically induced MSCs together with PRP. However, large placebo-controlled field studies are required in order to provide more insights in their regenerative capacities and *modus operandi*. Moreover, follow-up data after several years are necessary to confirm the sustainability of the repaired tissue.

## Conflict of Interest Statement

The author Jan H. Spaas declares competing financial interests as shareholder in Global Stem cell Technology (GST). Sarah Y. Broeckx and Jan H. Spaas are both employed by GST and inventors of several pending patents owned by GST (BE2012/0656; WO2014053418A9; WO2014053420A1; PCT/EP2013/075782). All other authors declare no conflicts of interests. The content of this manuscript contains a product under development owned by GST.
